# Steven Johnson Syndrome in a 102-Year-Old Woman in Saudi Arabia: A Case Report

**DOI:** 10.7759/cureus.32303

**Published:** 2022-12-07

**Authors:** Yasser H Alnofaiey, Wjood A AlTalhi, Wahaj A Altalhi, Abeer I Alsulaimani, Layla M Alkhaldi

**Affiliations:** 1 Department of Internal Medicine, Collage of Medicine, Taif University, Taif, SAU; 2 Family Medicine, Ministry of Health, Taif, SAU; 3 Medical Intern, Taif University, Taif, SAU

**Keywords:** emergancy, case report, saudi arabia, chronic kidney disease, steven-johnson syndrome

## Abstract

Medicines often cause serious immune-mediated mucocutaneous reactions including Steven-Johnson Syndrome (SJS) and toxic epidermal necrolysis (TEN). In the acute phase of SJS and TEN, a febrile illness is followed by cutaneous erythema with blister formation, skin and mucous membrane necrosis, and separation of the skin and mucous membranes. The patient swiftly becomes in danger of dying, necessitating immediate medical attention.

In this case report, we described a case of Steven-Johnson Syndrome in a 102-year-old female who was receiving palliative care and had stage 5 chronic renal disease. Although the agent that caused SJS in this patient is unknown, the patient was managed with topical medication, bandages for the lesions, and oral antihistamines. Skin biopsy, abdomen ultrasound, and sezary cell test were advised for the patient. Such presentations at that age have not, to our knowledge, been documented before.

## Introduction

Due to their high death rate, Stevens-Johnson syndrome (SJS) and toxic epidermal necrolysis (TEN) are severe bullous skin responses that are regarded as medical emergencies [1]. They are distinguished by erythema, hemorrhagic erosions, necrotic epidermal detachment, and mucocutaneous discomfort [1]. Both conditions are uncommon; the worldwide incidence of SJS and TEN, respectively, is 1-6 and 0.4-1.2 instances per million person-years [2]. The incidence of cutaneous medication responses is 8.259 percent, and they might vary from urticaria and erythema to SJS and TEN [3].

Serious immune-mediated mucocutaneous responses like SJS and TEN are often brought on by medicines [4]. Allopurinol, sulfonamide antibiotics, corticosteroids, oxicam, nonsteroidal anti-inflammatory medicines, chlormezanone, and nevirapine are only a few of the pharmaceuticals that have been implicated in the diseases' development [4-6]. The problems have also been connected to a few "over-the-counter" drugs including acetaminophen, metamizole, and ibuprofen [4, 7-8]. Roujeau et al. point out that protopathic bias may be to blame for the association between SJS and TEN and drugs such as acetaminophen, ibuprofen, and secretolytics [9]. HIV, the herpes virus, and mycoplasma pneumonia are among the illnesses that have been related to these disorders [10, 11].

A febrile illness is followed by cutaneous erythema with blister development, skin and mucous membrane necrosis, and separation of the skin and mucous membranes in the acute phase of SJS and TEN. The patient rapidly deteriorates into a life-threatening condition and is given emergency medical care [12].

The mortality result for patients may be impacted if they are not directed to a specialised area like an intensive care unit (ICU) or burns centre [13].

Despite the rarity of the aforementioned illnesses, they have terrible consequences for individuals who are afflicted. The respiratory and gastrointestinal systems may also be damaged, although cutaneous and ophthalmic issues are among the most frequent long-term consequences that have been found [14].

## Case presentation

A 102-years old female who had chronic kidney disease stage 5 with an estimated glomerular filtration rate of 4 mL/min/1.73m^2^ undergoing palliative treatment, presented with erythematous skin lesions all over the body with skin peeling. The patient was admitted on October 2021 with acute pulmonary edema for which she was on Mechanical Ventilator Milano (MVM) when her hemoglobin (Hb) levels were reduced and she received packed red blood cells (RBCs). Upon admission, the patient was suffering acute kidney injury (AKI) on top of CKD, for which she received hemodialysis once due to associated acute pulmonary edema. 

In June 2022, the patient underwent a fasciotomy in the forearm for the management of hematoma. Fasciotomy was done with four interrupted sutures, and dressing was done. Follow-up showed a clean wound. The patient later visited the emergency room (ER) on July 4, 2022, with a reported history of an itchy erythematous rash that started one month ago over her arms, hands, legs, and foot with skin peeling and decreased appetite. There was no history of fever, night sweats, rigors, change of level of consciousness, change of bowel habits, or history of similar conditions in the family.

By examination, the patient had a baseline level of consciousness, was vitally stable with no spikes of fever, and had a soft lax abdomen with a scaly rash over her arms, hands, legs, and foot. Her blood pressure was 110/80 mmHg, her heart rate (HR) was 76 bpm, and her O_2_ saturation was 94%. Laboratory investigations were done and the patient blood culture was positive for *Staphylococcus epidermidis*, nasal swab was positive for Methicillin-resistant *Staphylococcus aureus* (MRSA), and urine culture was positive for *Proteus mirabilis*. Her creatinine levels were 1180 mmol/L, CO_2_ pressure was 11 mEq/L, potassium level was 5.4 mmol/L, calcium level was 1.66 mmol/L, and phosphate was 2.4 mmol/L.

**Figure 1:**
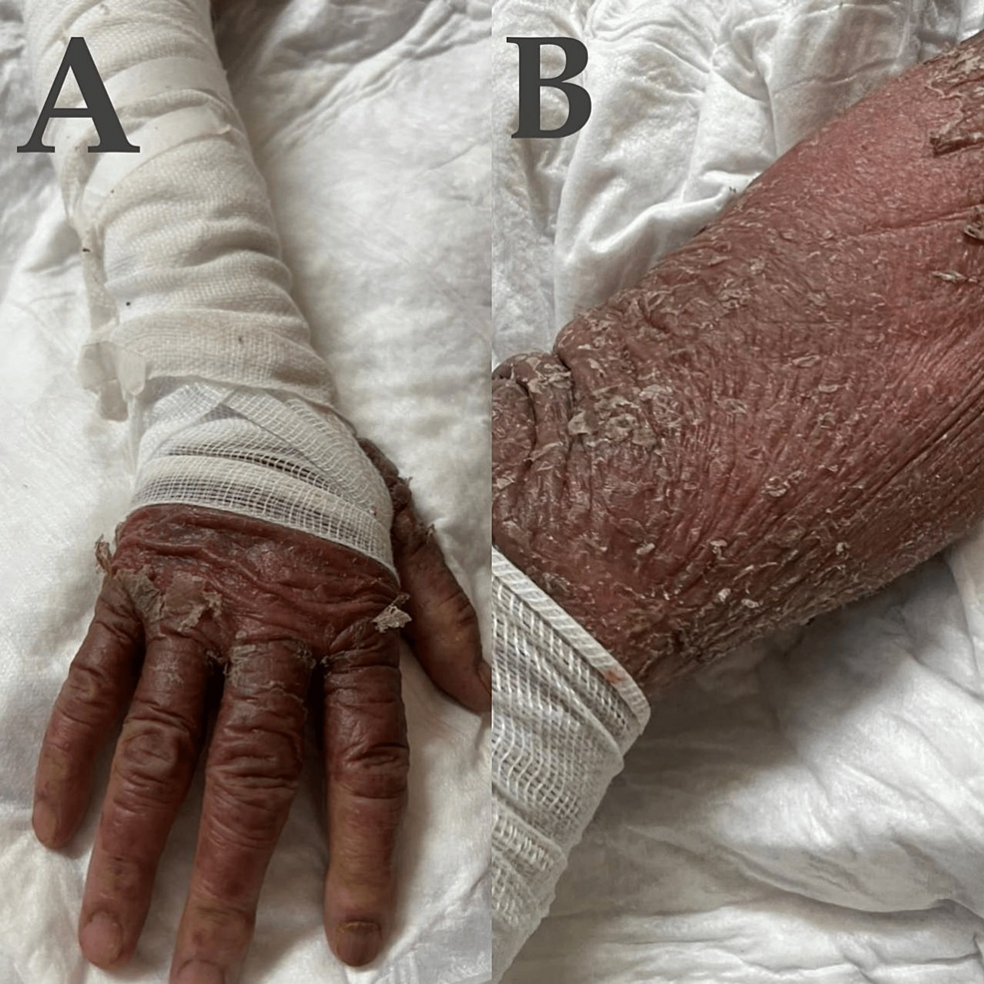
Dermatological manifestations of the patient. A, B : Painful itchy red hand skin that looks burned and peels off.

Assessment of the patient concluded that there were no signs of sepsis, and that the blood culture results are most likely caused by contamination of the sample. The patient was treated for cystitis with meropenem for five days. The dermatology team discharged her on cefuroxime. Skin lesions were assessed by the dermatology department (figure 1) two weeks before presentation to the ER and the patient was given cefuroxime, and when was seen again after two weeks, she was advised to do a skin biopsy.

**Figure 2:**
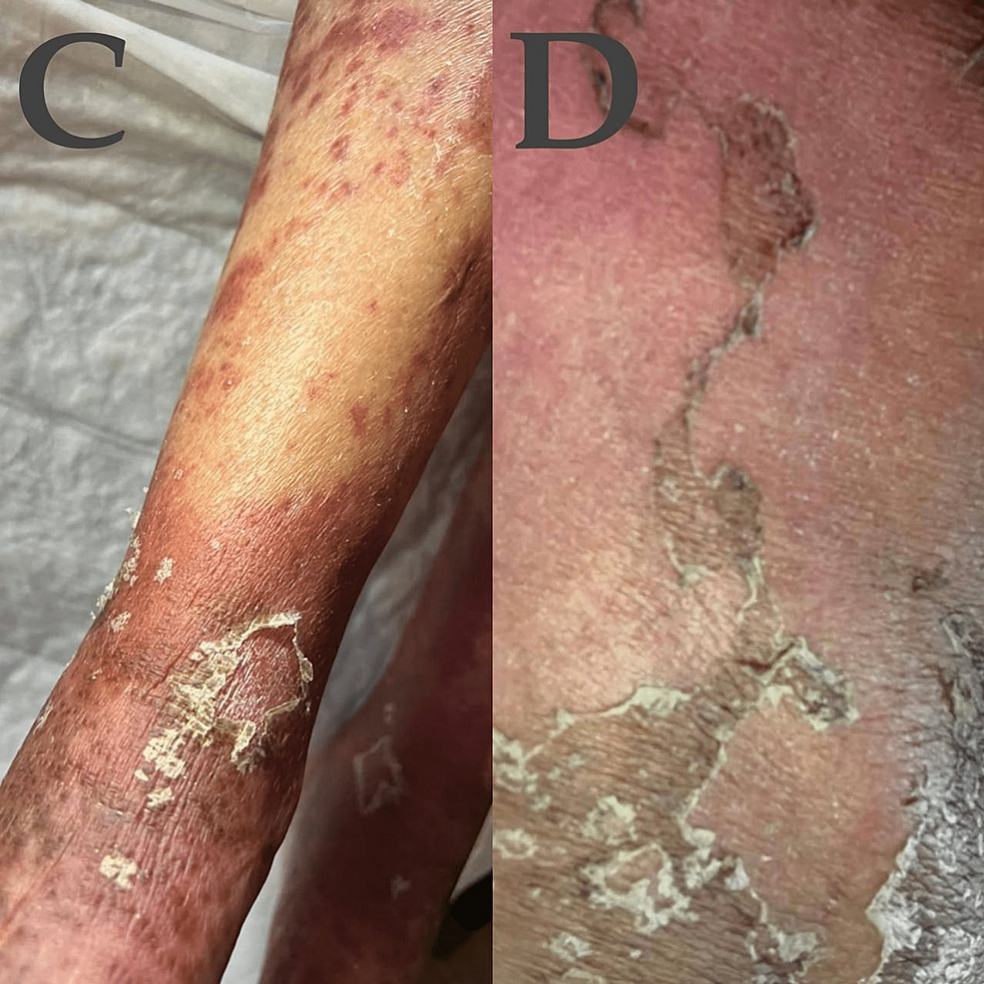
Dermatological manifestations of the patient C, D : Skin on the leg looks purple and peels off.

## Discussion

Given their hazards, side effects, and toxicity, the ease of access to medications and the population's unrestrained use of them are concerns related to public health. A rise in hospital admissions and mortality is caused by the harmful effects of drug usage and the incidence of adverse events. The term adverse drug reaction (ADR) refers to an unfavourable and unanticipated reaction to a medication that occurs at levels typically used for disease prevention, diagnosis, or treatment, or for the alteration of a physiological function [15].

The body surface area affected by SJS is less than 10% [5]. A transitional region between SJS and TEN is characterised by cutaneous detachment of 10% to 30%. TEN, often referred to as Lyell's syndrome, is characterised by epidermis detachment of more than 30% [16]. 

ADRs represent 17% of hospital admissions for the elderly. ADR was discovered to be the cause of 6.5% of hospital emergency admissions in England each year. The frequency of death from ADRs is thought to be 0.15% in the general population [17]. There are few prevalence statistics for Brazil [18]. The first signs of severe ADRs are often fever and malaise, which may continue or even become worse when lesions show up. They include stomatitis, balanitis, colpitis, acute conjunctivitis, and blepharitis, and are characterised by cutaneous erythema with blister development and hemorrhagic erosions of mucosal membranes [19]. Despite the seriousness of these illnesses, particularly TEN, there is currently no agreement on how best to treat patients, and there is a lot of variation in how SJS and TEN patients are treated. The fundamental component of therapy includes stopping the offending substance and providing supportive care [20].

In this case report, we described a case of Steven-Johnson Syndrome in a 102-year-old female that has stage five chronic kidney disease undergoing palliative treatment.

Data from the literature show that, contrary to what was discovered in a review of research, the incidence rises between the ages of 11 and 20 [21]. However, the proportion of senior patients who were hospitalised equals 6.98% of all cases that were recorded in a previous publication (n=86) [22].

The agent which caused SJS in this patient is not determined. However, the management lines of this patient were topical treatment and dressings for the lesions, and an oral antihistamine. The patient was recommended for sezary cell test, abdominal ultrasound, and skin biopsy.

Basic therapeutic effects include early response identification and medication withdrawal [23]. The major treatments for hospitalised patients were topical dressings for lesions, suspension of a suspected medicine, and systemic antibiotic therapy, sometimes after the onset of infectious problems. Along with serious eye lesions, the most pertinent consequences were infection of lesions and sepsis (including septic shock). The chance of late ocular lesions may be predicted based on the degree of systemic involvement by SJS [24].

## Conclusions

In conclusion, serious immune-mediated mucocutaneous responses like SJS are often brought on by medication and we would like to state that patients who started with any common drug may be at potential risk of developing SJS. A case of SJS in a 102-year-old female patient receiving palliative care for stage five chronic renal disease, to our knowledge, has not previously been reported at that age.
